# High Rate of Living Kidney Donation to Immigrant Children Despite Disparities—An Epidemiological Paradox?

**DOI:** 10.3389/fped.2019.00025

**Published:** 2019-02-12

**Authors:** Fatma Zehra Oztek-Celebi, Marion Herle, Valentin Ritschl, Lukas Kaltenegger, Tanja Stamm, Christoph Aufricht, Michael Boehm

**Affiliations:** ^1^Department of Pediatrics and Adolescent Medicine, Dr. Sami Ulus Obstetrics and Gynecology and Pediatrics Training and Research Hospital, Ankara, Turkey; ^2^Department of Pediatrics and Adolescent Medicine, Medical University of Vienna, Vienna, Austria; ^3^Section for Outcomes Research, Center for Medical Statistics, Informatics, and Intelligent Systems, Medical University of Vienna, Vienna, Austria

**Keywords:** pediatric renal replacement therapy, racial and ethnic disparities, preemptive transplantation, donor source, immigration

## Abstract

**Background:** Kidney transplantation is the preferred treatment modality for children with end-stage renal disease. In the adult population, migration-related modifiable factors were associated with low living donation rates; no such data are available on the pediatric population. This pilot study therefore compares donation modality, communication, knowledge, and attitudes/beliefs between families of immigrant and non-immigrant descent.

**Methods:** Demographic and clinical characteristics of a cohort of children from 77 families of immigrant (32; 42%) and non-immigrant (45; 58%) descent who had undergone renal transplantation were assessed and related to donation modality at the Medical University of Vienna. In a representative subset, modifiable migration-related factors were assessed in a questionnaire-based study.

**Results:** In immigrant families, information delay, limited communication, low knowledge levels, and self-reported conflicting beliefs were significantly more prevalent than in non-immigrants. The living kidney donation rate to children was high in both populations (immigrants: 63%, non-immigrants: 44%; *p* = 0.12). Living donation to children on dialysis was even significantly higher in immigrant families (immigrants: 13 out of 20; 57%, non-immigrants: 9 out of 33; 27%; *p* = 0.03).

**Conclusion:** Contrary to expectations, migration-related disparities did not translate into decreased living donation rates in immigrant families, in particular to children on dialysis. Certain factors might therefore be less important for the living donation process in pediatric care structures and/or might be overcome by yet undefined protective factors. Larger pediatric studies including qualitative and quantitative methods are required to validate and refine current conceptual frameworks integrating the perspective of affected families.

## Introduction

Kidney transplantation (KTx) is the treatment modality of choice in end-stage renal disease (1–3). Previously, racial and ethnic disparities in KTx have been reported in the pediatric setting (4). Children from minority groups in the US, particularly African-Americans, wait longer for a deceased donor (DD) KTx, and undergo less living donor (LD) and preemptive KTx compared to children of Caucasians (5–10). In Europe, a large ESPN/ERA-EDTA Registry report recently confirmed racial disparities in access to KTx (11), only few single center studies investigated the role of migration-associated disparities in pediatric KTx despite the increasing role of migration in Europe (12–15). Data from US immigrants are likely not transferable, as European immigrants are less likely to demonstrate racial differences, less likely to represent recent immigrants and mostly exposed to a less restrictive health care system (4, 11, 14, 15).

Recent research in the adult setting demonstrated that modifiable factors in the dimensions of communication, knowledge, and attitudes/beliefs are important in the living donation process (16). Based on that theoretical framework, two randomized trials successfully tested multicomponent interventions and resulted in increased referral of potential donors and actual LD KTx rates with stratified measures (17, 18). The same modifiable factors are also likely to differ between immigrant and non-immigrant families, and might therefore represent attractive targets to increase LD (and preemptive) KTx in that setting, thereby reducing disparities for children in immigrant families (4). To the best of our knowledge, however, neither disparities in modifiable factors (used to build the “adult” psychosocial concept of LD) nor their actual clinical relevance in immigrant and non-immigrant families on donation modality have been assessed in the pediatric setting of KTx to date.

The main objective of this pilot study was, therefore, to explore KTx donation modalities and modifiable factors in the dimensions of communication, knowledge, and attitudes/beliefs in parents of children with KTx in an Austrian cohort of families of immigrant or non-immigrant descent.

## Materials and Methods

### Participants and Survey

The study cohort consists of all families who were treated at the Medical University of Vienna between 2008 and 2013. The full cohort was used to establish baseline demographic and clinical characteristics; a random sample (the first 50 families who came to our transplant clinic during the study period) were asked to participate in the questionnaire-based modifiable factors study.

Demographic and clinical characteristics were taken from the patients' medical charts: age at KTx, sex, migration status, primary renal disease, first referral to pediatric nephrologist (FR), prior renal replacement therapy (RRT), preemptive vs. non-preemptive KTx, and organ donor source. A family's descent was classified as immigrant if the parents of the patient were first-generation immigrants (mother tongue of at least one parent was not German). In Austria, all patients have full insurance that cover all health-related costs, resulting in no disparities in listing and waiting times for KTx (14, 15). Patients who had already undergone transplantation were excluded.

### Study Questionnaire

Modifiable factors were evaluated in a questionnaire testing knowledge and assessing beliefs about LD KTx with support of one of the authors (FO-C; MB). If responding family members were uncertain about the meaning of a question, they were assisted and could ask for clarification. Out of 11 original belief statements published by Stothers (19), only six were used, since the remaining questions did not apply to a parent donor (“*Donating a kidney to someone requires an extremely close personal relationship”; “It is acceptable for a parent to receive a kidney from his/her child (over 18 years old)”; “Approaching a potential donor who then says no will change the relationship between the two people”; “Asking someone to donate makes the recipient seem selfish or greedy”; “Decisions about donation should be made by the donor alone. The recipient should not ask for a kidney”)*.

Questionnaires were prepared in Turkish, Serbian/Croatian and German, and validated using back-translation by native speakers of the target languages. After the back-translation had been completed, an extensive debriefing took place and the wording of the questionnaires was adapted.

### Statistical Analysis

A “knowledge score” was calculated by adding the number of correct answers for each individual on the seven items that focused on knowledge. If all statements were answered correctly, a maximum score of seven could be achieved. Responses to attitude/belief statements and donating experience were analyzed by assigning a score of 1 (for the least positive attitude toward donation) to 5 (for the most positive attitude toward donation); The scores for each statement were then added up to obtain a so-called “belief score” for each individual.

Variables were compared between groups using Wilcoxon Signed Ranks, Mann-Whitney-U, ANOVA and chi-square tests, as appropriate. A *P*-value of ≤ 0.05 was considered to be statistically significant. All analyses were performed with SPSS version 24. No power calculation was made. Bonferroni correction was not applied to the statistical tests as this study is of an exploratory nature and each test considered to be led by a separate, cross-sectional hypothesis.

### Ethical Consideration

The Medical University Vienna approved the study protocol [EK number 190/2008] and the study was carried out in line with the declaration of Helsinki. All participants were informed in detail about the study procedures and had to give oral and written informed consent.

## Results

### Baseline Characteristics and KTx Donation Modalities

Out of 77 children who received a kidney transplant and who were treated at the Medical University of Vienna from 2008 to 2013, 32 were from immigrant families (42%). Twenty-two families originated from ex-Yugoslavia (*n* = 12) or Turkey (*n* = 10), the rest (*n* = 10) from Romania, Bulgaria, Spain, Hungary, Jordan, Nigeria, Sudan, and Thailand. The baseline characteristics are shown in Table 1. There was no difference in the time from first referral to the start of RRT, the waiting time from listing at Eurotransplant to KTx, or age at KTx (Table 1).

**Table 1:** Baseline characteristics and information about renal replacement therapy.

	**Total cohort**				**Questionnaire subset**			
**Patients**	**Total (*n* = 77)**	**Immigrant (*n* = 32)**	**Non-immigrant (*n* = 45)**	***P***	**Total (*n* = 44)**	**Immigrant (*n* = 14)**	**Non-immigrant (*n* = 30)**	***P***
**Sex (*****n*** **= Female/Male)**	30/47	12/20	18/27	0.83^a^	17/27	6/8	11/19	0.69^a^
**Primary renal disease**				0.25^a^				0.34
CAKUT	38 (49%)	16 (50%)	22 (49%)		21 (48%)	5 (36%)	16 (53%)	
Cystic kidney disease	10 (13%)	6 (19%)	4 (9%)		6 (14%)	3 (21%)	3 (10%)	
FSGS	8 (10%)	1 (3%)	7 (16%)		5 (11%)	1 (7%)	4 (13%)	
Glomerulonephritis	5 (7%)	1 (3%)	4 (9%)		3 (7%)	0 (0%)	3 (10%)	
CNS	8 (10%)	5 (16%)	3 (7%)		7 (16%)	4 (29%)	3 (10%)	
Miscellaneous	8 (10%)	3 (9%)	5 (11%)		2 (5%)	1 (7%)	1 (3%)	
**Mean time from first referral to RRT (yrs)**	3.1 ± 3.4	2.4 ± 2.3	3.6 ± 3.9	0.6^b^	2.9 ± 3.2	2.4 ± 2.7	3.2 ± 3.4	0.88^b^
**First RRT**				0.35^a^				0.55^a^
KTx	21 (27%)	9 (28%)	12 (27%)		10 (23%)	3 (21%)	7 (23%)	
PD	32 (43%)	11 (34%)	21 (47%)		19 (43%)	5 (36%)	14 (47%)	
HD	17 (22%)	10 (31%)	7 (16%)		10 (23%)	5 (36%)	5 (17%)	
PD + HD	7 (9%)	2 (6%)	5 (11%)		5 (11%)	1 (7%)	4 (13%)	
**Mean age at KTx (yrs)**	8.4 ± 5.6	7.5 ± 5.6	9.1 ± 5.5	0.22^b^	7.4 ± 5.0	6.4 ± 4.6	7.9 ± 5.1	0.47^b^
**Mean waiting time**^***c***^ **(m)**								
All KTx	5.1 ± 6.2	3.9 ± 5.3	6.0 ± 6.7	0.15^b^	4.7 ± 6.3	2.6 ± 5.4	5.6 ± 6.6	0.07^b^
**DD KTx**	7.9 ± 6.0	7.6 ± 6.1	8.1 ± 6.1	0.69^b^	8.2 ± 6.8	7.3 ± 8.9	8.4 ± 6.6	0.39^b^
**LD KTx (% of total KTx)**	40 (52%)	20 (63%)	20 (44%)	0.12^a^	20 (46%)	10 (71%)	10 (33%)	0.02^a^

*RRT, Renal Replacement Therapy; CAKUT, Congenital Anomalies of the Urinary Tract; FSGS, Focal Segmental Glomerulosclerosis; CNS, Congenital Nephrotic Syndrome; ET, Eurotransplant; KTx, Kidney Transplantation; LD, Living Donor; DD, deceased donor; PD, Peritoneal Dialysis; HD, Hemodialysis, yrs, years; m, months*.

a *chi-square test*.

b *Mann-Whitney-U test*.

c *waiting time from listing to ET and date of KTx*.

**Figure 1:**
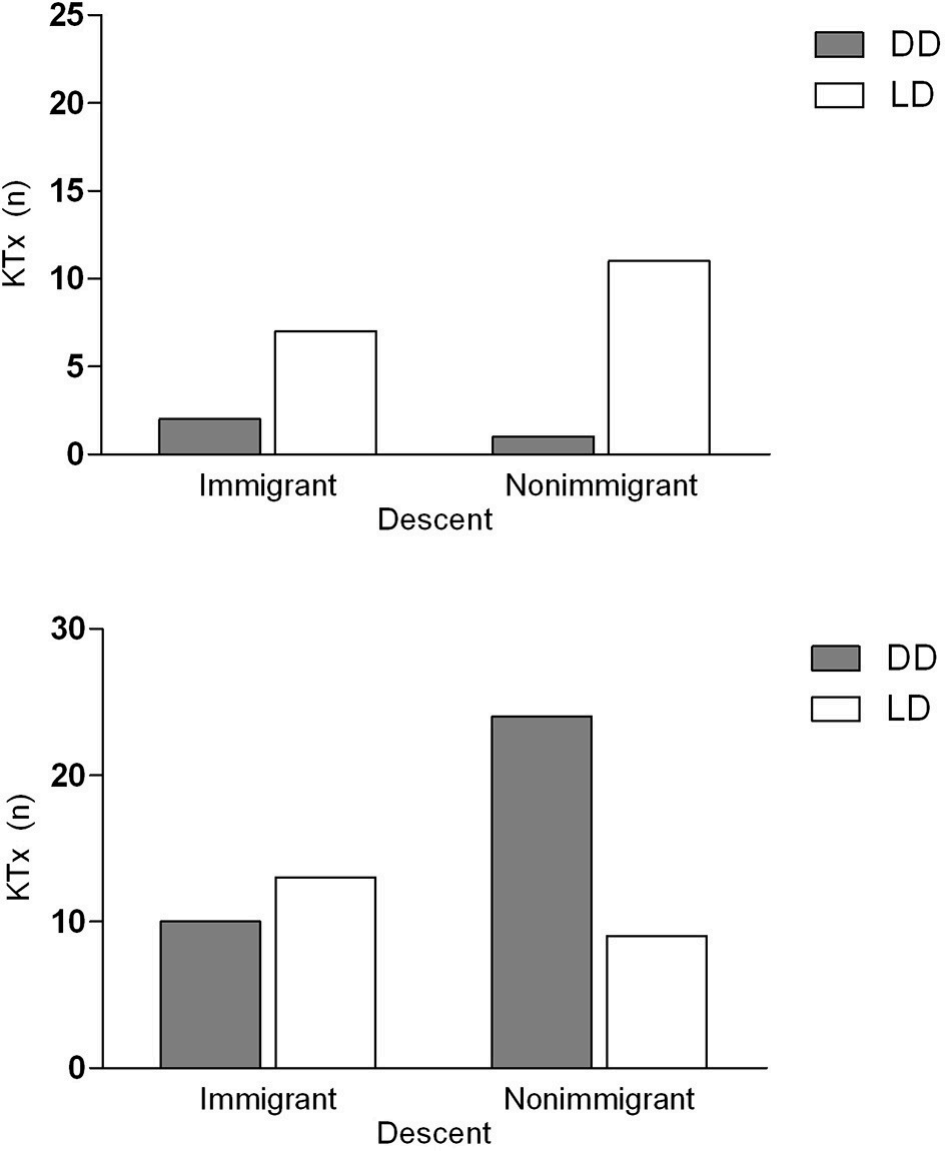
Kidney transplantation in families of immigrant and non-immigrant descent in Austria. Patients are stratified by donation modality of KTx (**upper panel**: preemptive, **lower panel**: non-preemptive) and by descent (left bars: immigrant and right bars: non-immigrant) and by donor source. The dark bars show the number of DD KTx, the light bars indicate the amount of LD transplantations. In non-preemptive KTx, the proportion of living donations was significantly higher in immigrant families than in non-immigrant ones (*p* = 0.03). KTx, Kidney transplantation; DD, deceased donor; LD, living donor.

Children from immigrant families tended to receive a LD transplant more frequently than non-immigrant ones (immigrant: 20 out of 32; 63%, non-immigrant: 20/45; 44%; *p* = 0.12). There were no differences in the overall rate of preemptive donation (immigrant: 9/32; 28%, non-immigrant: 12/45; 27%) (Table 1). In preemptive KTx, the proportions of LD were comparable (immigrant: 7/9; 78%, non-immigrant: 11/12; 92%; *p* = 0.37) (Figure 1). In non-preemptive KTx, the proportion of LD was significantly higher in immigrant families (immigrant: 13/20; 57%, non-immigrant: 9/33; 27%; *p* = 0.03) (Figure 1).

### KTx-Related Communication, Knowledge and Attitudes/Beliefs

Forty-four families (88% of those invited) agreed to participate in the questionnaire-based study, fourteen immigrant families and 30 non-immigrant ones (35 mothers, 9 fathers); out of these 23 children received a LD and 21 children a DD transplant. In 39 families, at least one parent was screened for feasibility of LD KTx. Twenty-three out of these 39 parents donated their kidney, significantly more from immigrant families (11/12) than from non-immigrant ones (12/27), (immigrant: 92%, non-immigrant: 44%; *p* = 0.009). As shown in Table 1, the questionnaire sub-population was representative of the total cohort regarding demographic and clinical characteristics.

KTx-related communication is demonstrated in Table 2. Both groups felt that they had enough time and sufficient information to consider organ donation (immigrant: 93%, non-immigrant: 90%; *p* = 0.8). However, significantly fewer immigrant families stated that the first communication on organ donation with the medical staff was 2 years or more before KTx (immigrant: 3 out of 14; 21%, non-immigrant: 16/30; 55%; *p* = 0.04). The medical staff were the main source of information on LD in both populations, but use of other sources of information, such as the mass media, to obtain information about organ donation was significantly lower in immigrant families (immigrant: 3/14; 23%, non-immigrant: 17/30; 57%; *p* = 0.04).

**Table 2:** Communication regarding living kidney donation in immigrant and non-immigrant families.

	**Immigrants (*n* = 14)**	**Non-immigrants (*n* = 30)**
**Did You Have Enough Time to Consider Living Kidney Donation?**^a^		
• Yes	12 (86%)	25 (83%)
• No	1 (7%)	1 (3%)
• Undecided	1 (7%)	2 (7%)
**Did You Have Sufficient Information On Living Kidney Donation For**		
**Your Decision?**		
• Yes	13 (93%)	27 (90%)
• No	0 (0%)	1 (3%)
• Undecided	1 (7%)	2 (7%)
**WHEN Was Your First Communication On Living Kidney Donation With The Medical Staff?**^b^		
• More than 2 years before the KTx ^c^	3 (21%)	16 (55%)
•1– 2 years before the KTx	6 (43%)	5 (17%)
• < 1 year before the KTx	5 (36%)	8 (28%)
**Who Suggested Living Kidney Donation To You?** ***(multiple answers are possible)***		
• Medical staff	10 (71%)	19 (63%)
• Family/relatives	1 (7%)	3 (10%)
• Myself	5 (36%)	10 (33%)
**Did You Have Alternate Sources Of Information Besides The Medical Staff?** ***(multiple answers are possible)***		
• media, journals, books ^*c*^	3 (21%)	17 (57%)
• Family/relatives	3 (21%)	3 (10%)

a *Two non-immigrant patients did not answer this question*.

b *One non-immigrant patient did not answer this question*.

c *p = 0.04*.

The knowledge results are shown in Table 3. The percentage of correct answers was significantly higher in the non-immigrant group (immigrant: 56%, non-immigrant: 70%; *p* = 0.045). A significantly higher percentage of immigrants than non-immigrants answered, “*I don't know”* to many of the knowledge statements (immigrants: 28%, non-immigrants: 12%; *p* = 0.03).

**Table 3:** Responses to knowledge statements (KS) in immigrant and non-immigrant families.

			**Correct**	**Incorrect**	**I don't know**	***P***
KS1	Kidney transplantation is preferred over dialysis for the treatment of kidney failure. (correct response: YES)	Immigrant	11 (84%)	1 (8%)	1 (8%)	0.882
		Non-immigrant	28 (94%)	1 (3%)	1 (3%)	
KS2	A person cannot spare a kidney since they are vital organs and are required for a healthy life. (correct response: NO)	Immigrant	11 (79%)	0 (0%)	3 (21%)	0.090
		Non-immigrant	27 (93%)	2 (7%)	0 (0%)	
KS3	Only immediate family members (brothers, sisters, parents, or children) can be living kidney donors. (correct response: NO)	Immigrant	9 (64%)	3 (21%)	2 (15%)	0.452
		Non-immigrant	23 (79%)	4 (14%)	2 (7%)	
KS4	Long-term health problems in living donors are very rare after kidney donation. (correct response: YES)	Immigrant	6 (50%)	4 (33%)	2 (17%)	0.368
		Non-immigrant	20 (69%)	7 (24%)	2 (7%)	
KS5	Immediate surgical side effects in donors are common and may be life-threatening. (correct response: NO)	Immigrant	9 (64%)	0 (0%)	5 (36%)	0.772
		Non-immigrant	22 (73%)	2 (7%)	6 (20%)	
KS6	Women may have difficulty with future pregnancies if they donate a kidney. (correct response: NO)	Immigrant	3 (22%)	2 (14%)	9 (64%)	0.040
		Non-immigrant	17 (57%)	6 (20%)	7 (23%)	
KS7	The success rates of living donor and deceased donor KT are about same. (correct response: NO)	Immigrant	4 (31%)	5 (38%)	4 (31%)	0.352
		Non-immigrant	6 (21%)	15 (52%)	8 (27%)	
Average Percentage^a^		Immigrant	56	17	28	0.04
		Non-immigrant	70	17	12	

a *Results for percentage are rounded numbers*.

The belief results are presented in Table 4. The total score for the belief statements did not differ between both groups, but two conflicting statements were more often chosen by immigrant families, “*since there is a life after death, people should enter the next life with a complete body”* and “*donating a kidney is a rewarding experience for donors”* (immigrant: 3/14; 21% and 11/13; 84%, non-immigrant: 1/28; 4% and 14/30; 48%; *p* = 0.04 and *p* = 0.03, respectively).

**Table 4:** Responses to belief statements (BS) in immigrant and non-immigrant families.

			**Agree**	**Disagree**	**I don't know**	***P***
BS1	It is ethically acceptable to take a kidney from a healthy person.	Immigrant	14 (100%)	0 (0%)	0 (0%)	0.902
		Non-immigrant	26 (92%)	1 (4%)	1 (4%)	
BS2	Since there is a life after death, people should enter the next life with a complete body.	Immigrant	3 (21%)	8 (58%)	3 (21%)	0.040
		Non-immigrant	1 (4%)	26 (92%)	1 (4%)	
BS3	Donors often agree to donate due to feelings of guilt or family pressure.	Immigrant	2 (15%)	9 (64%)	3 (21%)	0.872
		Non-immigrant	4 (14%)	19 (68%)	5 (18%)	
BS4	Donating a kidney is a rewarding experience for the living donors.	Immigrant	11 (84%)	1 (8%)	1 (8%)	0.030
		Non-immigrant	12 (44%)	5 (19%)	10 (37%)	
BS5	A living donor KT may strengthen the relationship between the donor and recipient.	Immigrant	11 (79%)	1 (7%)	2 (14%)	0.256
		Non-immigrant	15 (55%)	5 (19%)	7 (26%)	
BS6	Since the explantation of the kidney is not risk-free, someone who needs a KT should wait for a deceased donor kidney.	Immigrant	2 (14%)	6 (43%)	6 (43%)	0.123
		Non-immigrant	6 (22%)	17 (63%)	4 (15%)	

## Discussion

This study found disparities in communication and knowledge, and some significant differences in beliefs about LD KTx in immigrant families in Austria with children who had received a transplant. Surprisingly, these disparities were not associated with reduced access to LD KTx, but rather with a tendency toward a higher rate of living donation to children in immigrant families. Whereas, the preemptive donating rate was similar for both groups, significantly more LD KTx were performed in immigrant families than in non-immigrant ones, once dialysis had been initiated.

Our data describe clear differences in communication, knowledge, and beliefs in immigrant families in Austria, confirming findings regarding migration-related disparities in the adult setting of KTx (16). A delay in information on LD KTx from the medical staff, together with less use of the media, books, and other important sources of information on LD, might have resulted in a lower level of health literacy, exacerbated by limited access to reliable information in their mother tongues (20). Health literacy has previously been reported to be lower in immigrant families in other pediatric settings and associated with reduced access to LD KTx in the adult setting (21–23). However, health literacy was not directly assessed in our study, and alternate interpretations, such as positive effects on LD due to decreased exposure to misleading information from media might also be valid. Whether the lower knowledge levels concerning LD KTx in the immigrant families in our study, manifesting themselves in the form of more “*I don't know*” answers and more factually wrong answers such as “*Women may have difficulty with future pregnancies, if they donate a kidney*,” are attributable to reduced health literacy therefore needs clarification in future studies. In addition, higher prevalence of conflicting beliefs that might preclude or dissuade an individual from participating in LD such as “*since there is a life after death, people should enter the next life with a complete body*,” might pose a potential obstacle for LD in the immigrant families. Taken together, limitations in information/communication/knowledge about LD, combined with ambiguous attitudes/beliefs, are likely to increase decision-making conflicts regarding the living donation process (24).

However, our study found—in contrast to the expectations from the literature (25, 26)—no disparities in actual LD KTx rates in children of immigrant families in Austria. Why did migration-related factors that determined a low likelihood of LD in the adult setting (thus forming the current theoretical framework for LD) not result in reduced LD KTx rates in our pediatric population? The extent of the disparities in these modifiable factors, were almost identical in our immigrant group to those found in non-donors in the adult KTx population (19). In a previous study, we also found comparable disparities in measures of socioeconomic status, such as education levels and job quality, as was reported in the adult KTx populations (14, 19). We suggest as one possible explanation that adult and pediatric nephrology care settings are distinctly different in the context of the LD communication process. In the patient-centered adult setting, the patient with ESRD is expected to actively recruit potential donors. Consequently, deficits in communication with potential donors are directly linked to low rates of LD KTx (16). As a result, targeting this deficit with home-based education and communication by the transplant team was proven to be effective interventions (17, 18). In contrast, it is standard procedure in the family-focused pediatric setting for the interdisciplinary transplant team to repeatedly discuss all LD options with the parents, who inherently represent the most likely donors. In our study, this setting resulted in a high recruitment level, with about 90% of parents undergoing donor evaluation, compared to the adult setting with rates of <20% prior to intervention, and of about 60% post intervention (17, 18). Our results therefore suggest that certain migration-related disparities defined in the adult setting, such as limited communication and knowledge, might be less dominant in the case of the living donation process in pediatric care structures.

The surprisingly high rate of LD KTx in the immigrant families suggests that the disparities observed might have been more than overcome by yet undefined protective factors (16). The phenomenon of immigrant groups demonstrating equal or even better health outcomes than non-immigrants, despite the presence of obvious disparities, has previously been described as an “epidemiological paradox” (27). In the context of US-based KTx outcome studies, the “Hispanic Paradox” is used to summarize complex social and cultural “protective” factors such as strong ethnic identity and intense family relationships (9). Indeed, “*donating a kidney is a rewarding experience for donors”* was a significantly more frequently stated belief in the immigrant group, and medically adequate donors from immigrant families were twice as likely to ultimately donate a kidney than parents from non-immigrant families. Interestingly, the LD rate in immigrant families in Austria was selectively increased in non-preemptive KTx. Non-immigrant families appeared to be motivated to donate when it enabled a preemptive transplantation to be made but tended not to donate once the patient was on dialysis. In contrast, immigrant families were much more likely to donate when their child was on dialysis. The potential role of sociocultural factors for this apparently paradoxical donation pattern is supported by comparably high LD (with low preemptive donation) rates in Serbia and Turkey, the main countries of origin of immigrant families in Austria (26, 27). Unfortunately, our questionnaire was not designed to detect alternate explanations for the epidemiological paradox, such as concerns among the immigrant community that DD KTx may be less accessible to their group. To understand these findings better and to define as yet unknown factors, innovative study designs will be required which integrate qualitative methods in order to explore the perspective of the families affected of both immigrant and non-immigrant descent (28, 29).

This study has several limitations. Due to the rarity of pediatric KTx, the single center design precluded the use of complex statistical analysis and might have introduced bias of center specific experiences and attitudes of the pediatric nephrologists. For example, higher promotion of the advantages of LD could be a reason for the higher percentage of donor evaluation in our population. Our observations thus need independent validation to be generalizable to other populations or health care systems. However, it should be stated that the overall LD KTx rate in this study was comparable to the average European rate (26), suggesting that our results do not merely reflect an “Austrian-approach” effect. Small numbers of responders may underestimate difference in the complex psychosocial setting of LD in chronic renal failure, therefor a multicenter study should be envisaged. However, this was an exploratory study that yielded unexpected new findings in a representative pediatric cohort. Parents who participated in the questionnaire subgroup might have differed from those who did not. However, no significant differences were noted in terms of demographic or clinical variables. As the study was performed in families with children who had already undergone KTx, data on beliefs and knowledge were subject to several biases, including *post-hoc* justification and recall bias. However, their responses best reflect their current perception of the situation having had time to reflect. Despite these shortcomings, our pilot study delineated—in addition to the novel observation of an apparently paradoxical donation pattern in immigrant families—important differences to current adult concepts of the LD process.

In conclusion, our study describes high rates of pediatric LD KTx in immigrant families in Austria, despite confirmation of significant disparities, such as information delay, limited communication, lower knowledge levels, and more conflicting belief systems regarding LD. Therefore, certain migration-related disparities defined in the adult setting might be less important/dominant in the case of the living donation process in pediatric care structures and did not translate into reduced willingness to donate. The observation that immigrant families were much more likely to donate once they saw the challenges of dialysis (for patient and/or family) additionally suggests yet undefined migration-related protective factors (“epidemiological paradox”). These new and unexpected findings of our pilot study indicate the need for future quantitative and qualitative research to validate this donation pattern in independent populations and to explore yet unknown perspectives of immigrant and non-immigrant families to build a robust theoretical framework for pediatric KTx.

## Author Contributions

All authors have significantly contributed to conception (FO-C, CA, MB), design (FO-C, CA, MH, MB), analysis (FO-C, LK, CA, MB) and interpretation of data (FO-C, CA, MH, VR, TS, LK, MB). The questionnaires were filled out during a questionnaire-based study with FO-C and MB. All authors gave final approval of the submitted version. The results presented in this paper have not been published previously in whole or part, except in abstract form.

### Conflict of Interest Statement

The authors declare that the research was conducted in the absence of any commercial or financial relationships that could be construed as a potential conflict of interest.
